# CHANGING TALK (CHAT) ONLINE COMMUNICATION EDUCATION FOR ADULT DAY CARE STAFF: EVALUATION AND FUTURE DIRECTIONS

**DOI:** 10.1093/geroni/igac059.1614

**Published:** 2022-12-20

**Authors:** Kristine Williams, Carissa Coleman, Maria Hein, Clarissa Shaw, Yelena Perkhounkova, Tim Beachy

**Affiliations:** University of Kansas Medical Center, Lawrence, Kansas, United States; University of Kansas, Kansas City, Kansas, United States; University of Iowa College of Nursing, Iowa City, Iowa, United States; University of Iowa, Iowa City, Iowa, United States; University of Iowa, Iowa City, Iowa, United States; University of Iowa, Iowa City, Kansas, United States

## Abstract

Communication education has been effective in reducing elderspeak use for nursing home staff and subsequently reduces resistiveness to care and inappropriate antipsychotic medication use in residents living with dementia. Communication is fundamental for dementia care and educating staff in other long-term service and support settings such as Adult Day Care (ADC) is needed. Tailoring education to the specific setting is indicated to optimize acceptability and effectiveness. ADC staff (N=22) from 3 ADC centers participated in the Changing Talk: Online Training (CHATO). Results for the 19 staff who completed the education program were analyzed. A focus group was conducted with a subset of participants to evaluate feasibility, applicability, and directions for tailoring for ADC settings. Knowledge gain was demonstrated by score increases 15.0 percentage points (p<.001) from pre- to post-education on the Changing Talk Scale (CHATS). Confidence in Dementia Care (CODE) scores significantly increased after CHATO (p=.05), indicating an increase in confidence in caring for clients with dementia. The mean Modified Diffusion of Innovation scale score was 2.2 (1=intend to use skills in practice to 5=don’t intend to use), indicating intent to use with clients. Program evaluation ratings were positive indicating satisfaction. Focus group participants reported that the CHATO was valuable and recommended ADC specific scenarios. The results support the value of CHATO for ADC staff as well as specific directions to better tailor the education to increase applicability in this LTSS setting.

